# A Novel Co-polymer Based on Hydroxypropyl α-Cyclodextrin Conjugated to Low Molecular Weight Polyethylenimine as an *in Vitro* Gene Delivery Vector

**DOI:** 10.3390/ijms9112278

**Published:** 2008-11-21

**Authors:** Hongliang Huang, Hai Yu, Da Li, Yang Liu, Fenping Shen, Jun Zhou, Qingqing Wang, Guping Tang

**Affiliations:** 1 School of Life Science, Guangdong Pharmaceutical University, Guangzhou Higher Education Mega Center, Guangzhou, P. R. China. E-Mail: honglianghuangcn@hotmail.com; 2 Institute of Immunology, Zhejiang University, Yuhangtang Road 388, Hangzhou, P. R. China. E-Mails: yuhai@zju.edu.cn (H. Y.); lidaonco@yahoo.com.cn (D. L.); liuyang1006@163.com (Y. L.); shenfenping@yahoo.com.cn (F. S.); 3 Institute of Chemical Biology and Pharmaceutical Chemistry, Zhejiang University, Tianmushan Road 148, Hangzhou, P. R. China. E-Mail: zerobomp@zju.edu.cn (J. Z.)

**Keywords:** Polyethylenimine, 2-hydroxypropyl-α-cyclodextrin, non-viral gene delivery vector, co-polymer

## Abstract

A novel co-polymer based on 2-hydroxypropyl-α-cyclodextrin cross-linked by low molecular weight polyethylenimine was synthesized as a gene delivery vector. The copolymer could bind and condense DNA tightly. It showed lower cytotoxicity than PEI 25kDa in SK-BR-3 cells. Transfection efficiency was increased over 5.5-fold higher than PEI 25 kDa in SK-BR-3 cells in complete serum medium. It is a potential candidate vector for gene therapy.

## 1. Introduction

Efficient and safe vectors are a critical element for DNA delivery in gene therapy. Viral vectors have been used as delivery vectors in most gene therapy methods because of their very efficient gene delivery, but they suffer from problems such as safety concerns [1], limited loading capacity and limited scale of production [2]. Non-viral vectors have some advantages over viral approaches. A good non-viral gene delivery vector should display low cytotoxicity and high efficiency of DNA delivery into cells [2]. Polyethylenimine (PEI) is a cationic polymer in wide usage in research as a non-viral gene delivery vector. Its transfection efficiency and toxicity depend on its molecular weight – high molecular weight PEI showed high transfection efficiency, and low molecular weight PEI (less than 2000 Da) showed low transfection efficiency and low toxicity [3–5]. Petersen *et al*. significantly enhanced the plasmid DNA condensation of low molecular weight (LMW) PEI by cross-linking it with star-shaped polyethylene [6]. Cyclodextrins (CDs) are cyclic oligomers of glucose, which are biocompatible and have low toxicities in animals and humans [7]. CDs had been incorporated into cationic polymers which have been employed for gene delivery since 1999 [8–11]. Cationic polymers modified with CDs showed lower cytotoxicity and higher gene transfection [12–14]. Tang *et al*. synthesized cationic polymers by using β-CD to cross-link PEI (Mw 600), which displayed high transfection efficiency compared to PEI 25 kDa and effectively mediated gene transfection in the central nervous system [15]. Yang *et al*. synthesized a novel cationic star-shaped polymer as a non-viral gene delivery vector by conjugating PEI to α-CD. The star-shaped polymer showed excellent transfection efficiency *in vitro* [16]. In our previous study, hydroxypropyl-β-CD (HP β-CD) and hydroxypropyl-γ-CD (HP γ-CD) were linked to LMW PEI as gene delivery vectors. The co-polymers had low cytotoxicity and high gene transfection efficiency properties in SKOV-3 cells [17]. In this study, a novel non-viral vector based on 2-hydroxypropyl-α-cyclodextrin (HP α-CD) conjugated to LMW PEI is presented. The structure was confirmed by ^1^H-NMR. Its cytotoxicity and gene transfection efficiency were measured in SK-BR-3 cells.

## 2. Results and Discussion

**Scheme 1.**
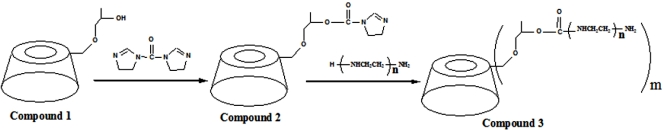
Synthesis route of HP α-CD-PEI.

The co-polymer was synthesized as shown in Scheme 1. The method has been described in the literature [17].

Compound 1 is HP α-CD, Compound 2 is HP α-CD-CDI, Compound 3 is HP α-CD-PEI (n = 14, m = 3.6)

### 2.1. ^1^H-NMR of the co-polymer

**Figure 1.**
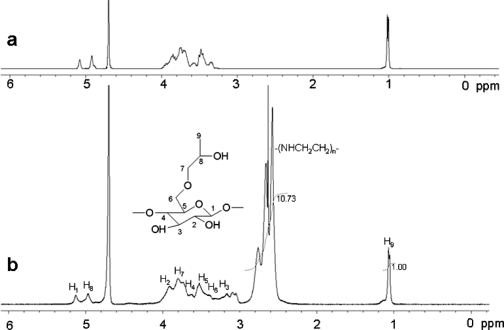
^1^H-NMR spectra of (a) HP α-CD, (b) HP α-CD-PEI.

The structure of HP α-CD-PEI was confirmed by ^1^H-NMR (Figure 1). The signal at δ 1.038 p.p.m. was assigned to the -CH_3_ protons of the HP α-CD hydroxypropyl group, and the signals at δ 2.4–3.0 p.p.m. were assigned to the -NHCH_2_CH_2_- protons from the PEI 600 Da. From the 1H-NMR integration values it could be estimated that the ratio of CD and PEI was 1:10.7.

### 2.2. Particle size and zeta potential test

**Figure 2.**
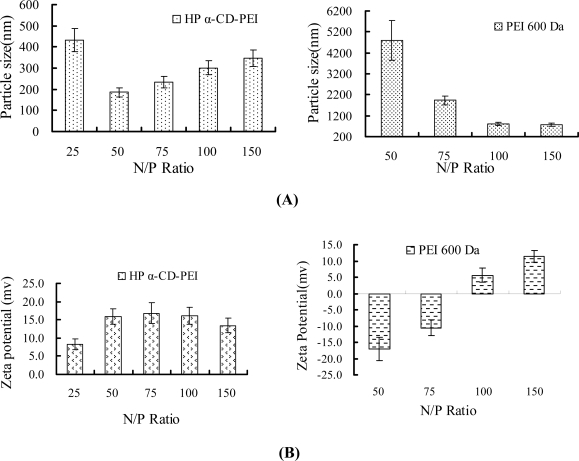
(A) Particle size (B) Zeta potential of HP α-CD-PEI/DNA and PEI 600 Da/DNA complexes.

Figure 2 shows the particle size and zeta potential of HP α-CD-PEI/DNA and PEI 600/DNA complexes. When N/P ratio was at 50 (N/P ratio, the number of nitrogen residues for PEI per the number of phosphate for DNA), the particle size of HP α-CD-PEI/DNA/DNA complexes was close to 186 nm, and that of the PEI/DNA complexes was about 4,800 nm. The zeta potential of HP α-CD-PEI/DNA complexes was about +16.0 mV, and PEI 600/DNA was about -17 mV. The positively charged polymer could condense efficiently with negatively charged DNA, but it would aggregate *in vivo* and *in vitro*. In our experiment, the zeta potential of HP α-CD-PEI/DNA complexes was about +16.0 Mv, lower than PEI 25 kDa, which would avoid aggregation (the zeta potential of PEI 25 kDa was about +27.4 mV when N/P Ratio was at 10; data shown in the Supplementary Material). We thought that after the CD conjugated with PEI 600 to form the co-polymer, it could efficiently compact DNA into nano-particles. The improvement of condensing DNA with LMW PEI might be attributed to the formation high molecular weight co-polymer.

### 2.3. Agarose gel electrphoresis assay

**Figure 3.**
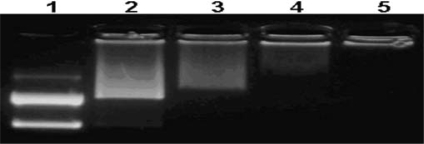
Agarose gel electrophoresis retardation of pGL3 plasmid DNA by HP α-CD-PEI. Lane numbers correspond to HP α-CD-PEI/DNA at different N/P ratios: (1) 0:1 (DNA only), (2) 1:1, (3) 2:1, (4) 4:1, (5) 6:1.

Figure 3 shows the capacity of the co-polymer to condense DNA. The migration of DNA was completely retarded when the N/P ratio of co-polymer/DNA was at 4:1.

### 2.4. Cell viability assay

**Figure 4.**
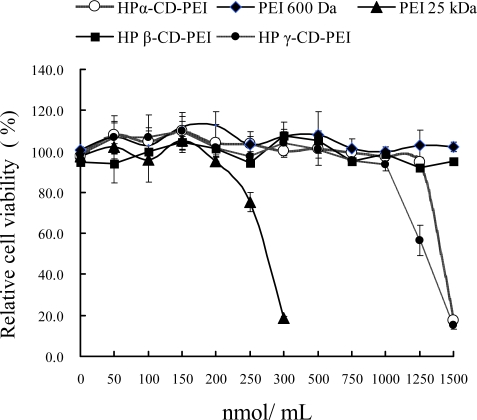
Cell viability of co-polymer/DNA complexes in SK-BR-3 cells.

Figure 4 shows the cell viability assay results for different co-polymer/DNA complexes in SK-BR-3 cells. For HP-α-CD-PEI, when the concentration was up to 1,250 nmol/mL, the cell viability was about 94.7 ± 2.4%. For PEI 25 kDa, cell viability was only 18.6 ± 0.7% when the concentration was 300 nmol/mL. For HP γ-CD-PEI group, cell viability was about 15.3 ± 1.9% when the concentration was 1,500 nmol/mL. For HP β-CD-PEI group and PEI 600, they maintained low toxicity at all concentrations. The results indicated that HP α-CD-PEI and HP γ-CD-PEI have low cytotoxicity and HP β-CD-PEI has no cytotoxicity in SK-BR-3 cells.

### 2.5. In vitro gene delivery by co-polymer/DNA complexes

**Figure 5.**
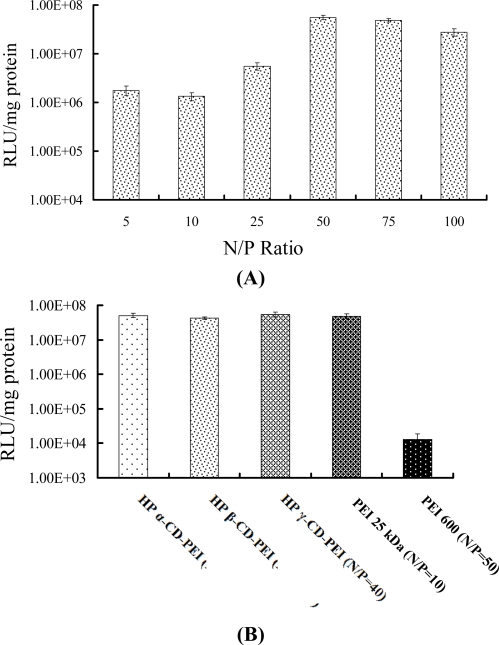
Luciferase assay of co-polymer/DNA complexes in SK-BR-3 cells. **(A)** HP α-CD-PEI/DNA complexes at different N/P ratios, **(B)** HP α-CD-PEI/DNA complexes (N/P ratio is 50), HP β-CD-PEI/DNA complexes (N/P ratio is 300), HP γ-CD-PEI/DNA complexes (N/P ratio is 40) and PEI 25 kDa/DNA complexes (N/P ratio is 10).

Figure 5 shows the co-polymer/DNA complex transfection efficiency results in SK-BR-3 cells. The results explored the optimal N/P ratio of co-polymer/DNA complexes for *in vitro* gene delivery. For HP α-CD-PEI/DNA complexes the optimal N/P ratio was at 50: 1 (Figure 5 A). For HP β-CD-PEI/DNA and HP γ-CD-PEI/DNA complexes the optimal N/P ratios were at 300:1 and 40:1 (data shown in the Supplementary Material). The Relative Light Unit data (RLU/mg protein) for HP α-CD-PEI was 5.1 × 10^7^, which was close to that of PEI 25 kDa (5.0 × 10^7^). It was 3,930-fold higher than that of PEI 600 Da at N/P ratio of 50.

**Figure 6.**
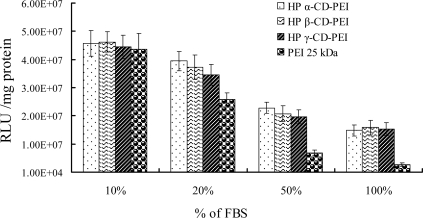
Transfection efficiency of polymer/DNA complexes (at N/P ratio of 50) in different FBS concentrations medium in SK-BR-3 cells.

Figure 6 shows the results of transfection efficiency of co-polymers in different FBS (Fetal Bovine Serum) concentrations of culture mediums. The serial concentrations were 10%, 20%, 50% and 100%, respectively. In 10% FBS medium the RLU reading of HP α-CD-PEI was 4.7 × 10^7^, or 1.05-fold higher than PEI 25 kDa (4.4 × 10^7^), Increased the concentration of FBS, from 20% to 100%, they were 1.5, 3.3 and 5.5 times higher than PEI 25 kDa, respectively.

**Figure 7.**
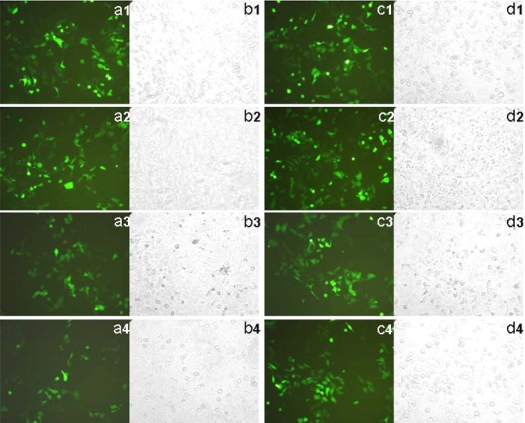
Fluorescent and bright field images of enhanced green fluorescent protein expression in SK-BR-3 cells transfected with of PEI 25 kDa carrying pEGFP at N/P ratio of 10 in different FBS concentration medium (a1, in 10% FBS; a2, in 20% FBS; a3, in 50% FBS; a4, in 100% FBS), HP α-CD-PEI carrying EGFP plasmid at N/P ratio of 50 (c1, in 10% FBS; c2, in 20% FBS; c3, in 50% FBS; c4, in 100% FBS). Image of column a1 to a4 and c1 to c4 were in fluorescence, image of column b1 to b4 and d1 to d4 were in bright field.

Figure 7 shows the transfection efficiency of HP α-CD-PEI/pEGFP (plasmid of enhanced green fluorescent protein) in SK-BR-3 cells. PEI 25 kDa was used as control. Figures 7a1 to Figure 7a4 correspond to PEI 25 kDa/DNA groups in different concentration of FBS (10%, 20%, 50% and 100%, respectively). Figures 7c1 to Figure 7c4 were the HP α-CD-PEI/DNA group. From the images we could infer that the positive cells which expressed GFP (green fluorescence protein) in PEI 25 kDa and HP α-CD-PEI had no significant difference at low concentrations of FBS (10% and 20% ). Increasing the concentration of FBS, the expressed GFP were decreased in two polymers. But the cells transfectd with HP α-CD-PEI expressed much more fluorescent protein than that of PEI 25 kDa in 100% FBS.

**Figure 8.**
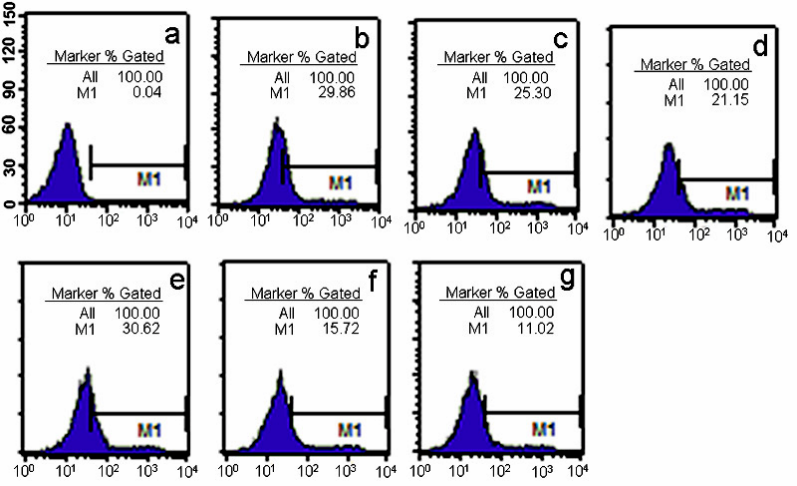
Flow cytometric analysis of pEGFP expression in SK-BR-3 cells using HP α-CD-PEI (N/P ratio at 50) and PEI 25 kDa (N/P ratio at 10). **(a)** Cells of control (untransfected), (b-d) HP α-CD-PEI (b, in 10% FBS; c, in 50% FBS; d, in 100% FBS), (eg) PEI 25 kDa(e, in 10% FBS; f, in 50% FBS; g, in 100% FBS).

Figure 8 shows the results of flow cytometric analysis using HP α-CD-PEI/DNA and PEI 25 kDa/DNA complexes in SK-BR-3 cells. The images indicate that the ratio of green fluorescence positive cells using HP α-CD-PEI/DNA complexes (Figure 8c) was 1.61-fold higher than that of PEI 25k Da/DNA complexes (Figure 8f) in 50% FBS medium, it (Figure 8d) was 1.91-fold higher than PEI 25 kDa/DNA complexes (Figure 8g) in 100% FBS medium, it (Figure 8b) was close to PEI 25 kDa/DNA complexes (Figure 8e) in 10% FBS medium.

Cyclodextrin can stimulate cholesterol efflux from cultured cells with high efficiency [18–19]. Zhao *et al*. [20] used CDs to increase the cellular uptake of phosphorothioate oligodeoxynucleotides. Cyclodextrins can enhance their gene transfer efficiencies when they were used together with nonviral gene carriers [21–22]. In our study, a novel co-polymer based on LMW PEI linked to HP α-CD was synthesized, and it showed low cytotoxicity and high transfection efficiency. Especially in high concentration of FBS, the co-polymer showed higher transfection efficiency than that of PEI 25 kDa in SK-BR-3 cells. A plausible explanation was that the zeta potential of co-polymer/DNA complexes was lower than that of PEI 25 kDa and as a result, it could bind more cell membranes than protein in serum. Another explanation was that cyclodextrins can improve the cellular delivery of oligonucleotides [7]. The results suggested the co-polymer should be promised candidate for *in vitro* and *in vivo* gene delivery.

## 3. Experimental Section

### 3.1. Materials and cells

PEI (MW 600 Da and MW 25 kDa), HP α-CD and (3-(4,5-dimethylthiazol-2yl)-2,5-diphenyl tetrazolium bromide (MTT) were purchased from Sigma-Aldrich. CDI was purchased from Pierce Corporation. SK-BR-3 cells (Human breast cancer cell line) was purchased from American Type Culture Collection (Rockville, MD). Cells were cultured in RPMI 1640 supplemented with 10% FBS (Gibco) and 1% antibiotic (penicillin-streptomycin, GibcoBRL) in a 37 °C incubator with 5% CO_2_.

### 3.2. Plasmid preparation

The plasmid pGL3-Luc encoding luciferase was purchased from Promega (Madison, WI). The pc DNA 3.1 pEGFP (encoding enhanced green fluorescence protein, EGFP) was a gift from the Institute of Immunology, Second Military Medical University, Shanghai, P.R. China. All plasmids were amplified in E. *coli* and purified according the supplier’s protocol (Mega Endofree Plasmid Purification Kit, Qiagen, Hilden, Germany).

### 3.3. ^1^H-NMR Measurements

^1^H-NMR spectra of samples (10 mg HP α-CD and HP α-CD-PEI samples in 0.7 mL D_2_O) were recorded on a Varian 400MHz spectrometer (32 scans at room temperature).

### 3.4. Particle size and zeta potential test

HP α-CD-PEI/DNA and PEI 600/DNA complexes were prepared at a DNA concentration of 30 μg/mL with N/P ratio of 25, 50, 75, 100, and 150, respectively, in 150 mmol/L NaCl. Size and zeta potential of polymer/DNA at different N/P ratios were measured with 90Plus/BI-MAS (Brookhaven Instruments Corporation) at room temperature. Scattering light was detected at 90°angle, running of 200 sec for each sample and analyzed in the Unimodal Analysis mode.

### 3.5. Agarose gel electrphoresis assay

The plasmid DNA (pGL3) was 0.5 μg in 1 μL of TE buffer. The amount of co-polymer added was calculated based on a designed N/P ratio of co-polymer/DNA. The formed co-polymer/DNA complexes were mixed with a loading buffer and loaded onto 1% agarose gel containing Ethidium Bromide. Gel electrophoresis was run at room temperature in TAE buffer (1×) at 80 V for 40 min. DNA bands was visualized by a UV (254nm) illuminator.

### 3.6. Cell viability assay

SK-BR-3 cell was employed to investigate the toxicity of co-polymers. The different copolymer/DNA complexes were prepared in serum supplemented cell culture medium. The cells (2×10^4^ cells/well) were seeded into 96-well plates (Costar, Corning Corp. New York). After overnight incubation the culture medium was replaced with 100 μL serial dilutions of the co-polymer/DNA complexes in fresh medium, the serial concentration of the polymers were 50, 100, 150, 200, 250, 300, 500, 750, 1000, 1250, 1500 nmol/mL (the corresponding N/P ratios were 7, 14, 21, 29, 36, 43, 71, 107, 143, 179, 214), then the cell was incubated for another 4 h. Sterilized MTT in PBS was added to each well reaching a final concentration of 0.5 mg MTT/mL. After 4 h, un-reacted dye was removed by aspiration. The crystals were dissolved in 100 μL/well DMSO and measured spectrophotometrically in an ELISA reader (Model 680, Bio-Rad) at a length of 570 nm. The relative cell growth (%) related to controls containing cell culture medium without co-polymer was calculated by test/control×100%.

### 3.7. Transfection efficiency with luciferase assay

SK-BR-3 cell was seeded in 24-well plates (Corning, Costar) in 0.75 mL of fresh RPMI 1640 supplemented with 10% FBS containing antibiotics 24 h prior to transfection. pGL3 (1 μg/well) was used for transfection. The medium was replaced with fresh RPMI 1640 (0.5 mL) without FBS or supplemented with 10%, 20%, 50% and 100% FBS. PEI 600 Da/DNA, PEI 25 kDa/DNA and different co-polymers/DNA complexes were incubated with the cells for 4 h. Then the medium was replaced with fresh RPMI 1640 (0.75 mL) with 10% FBS and cells were further incubated for 36 h. Cells were permeabilized with 100 μL of cell lysis buffer (Promega Corporation). The luciferase activity in cell extracts was measured using a luciferase assay kit (Promega) on a single-well luminomiter (Berthold lumat LB9507, Germany) for 10 sec. The relative light units (RLU) were normalized against protein concentration in the cell extracts, which was measured using a BCA protein assay kit (Pierce, Rockford, USA).

### 3.8. Transfection efficiency with fluorescence microscope

The plasmid DNA (pEGFP) in the amount of 1 μg per well was employed for transfection. When the cells were transected, the medium in each well was replaced with fresh RPMI 1640 (0.5 mL) with 10%, 50% and 100% FBS. Co-polymer/DNA complexes (at N/P ratio of 50) were incubated with the cells for 4 h. Then the medium was replaced with fresh RPMI 1640 (0.75 mL) with 10% FBS and cells were further incubated for 36 h. After the incubation, cells were observed in fluorescence microscope. The fluorescence photographs of GFP positive SK-BR-3 cells were taken by a Leica (DMLB&DMIL) microscope equipped with Digital 1/2 inch CCD and Leica MPS 60. The optimal exposure time was 5 sec.

### 3.9. Flow cytometry analysis

Cell was trypsinized, washed and re-suspended in PBS. FACS analysis of EGFP-expressing cell was performed on a Flow Cytometry (BD LSR, USA), according to their green fluorescent emission. Untransfected cells were used to set the background.

### 3.10. Statistics analysis

The data were submitted to Normality and Equal Variance tests, which revealed normal distribution. Statistical analysis was made using ANOVA and a multiple comparisons test. Statistical calculations were computed with a statistic software package (SPSS 10.0). For all tests, the results are expressed as the mean±s.d. A mean with P 0.05 was considered statistically significant.

## 4. Conclusions

A novel co-polymer based on LMW PEI linked to 2-hydroxypropyl-α-cyclodextrin was synthesized. This co-polymer showed low cytotoxicity and high transfection efficiency in SK-BR-3 cells. Especially in high concentration of FBS it showed higher gene transfection efficiency than PEI 25 kDa. The results were confirmed by luciferase assay, fluorescence microscope and flow cytometry analysis. All of it suggested it should be a promised candidate carrier for *in vitro* and *in vivo* gene delivery.
